# O29 Keeping a watchful eye: IgG4-positive lymphoma masquerading as IgG4-related ocular disease

**DOI:** 10.1093/rap/rkab067.028

**Published:** 2021-10-19

**Authors:** Yik Long Man, Charlie Hession, Geoffrey Rose, Bridget Wilkins, David Wrench, David D'Cruz

**Affiliations:** 1 Guy's & St Thomas' NHS Foundation Trust, London, United Kingdom; 2 Moorfields Eye Hospital NHS Foundation Trust, London, United Kingdom

## Abstract

**Case report - Introduction:**

IgG4-related disease (IgG4-RD) is increasingly recognised in rheumatology practice. We describe a case of orbital swelling initially diagnosed as IgG4-RD. It was exquisitely steroid responsive and she was treated with 6 years of immunosuppression but her symptoms later deteriorated. She developed worsening left eye swelling that required debulking surgery; histopathology demonstrated IgG4-positive lymphoma. She had an excellent response to localised radiotherapy and remains in remission. Although her initial presentation was typical of IgG4-RD, her subsequent lack of response to steroids prompted reconsideration of the initial diagnosis and re-examination of her previous biopsy specimen, confirming IgG4-positive Iymphoma at both timepoints.

**Case report - Case description:**

A 25-year-old lady presented in 2008 with a swollen left eye. She had a history of childhood eczema, asthma and sinusitis. Diagnostic excision was undertaken; histology was considered to show chronic inflammation, scattered eosinophils, dense fibrosis and no granulomas. She was diagnosed with a ‘granulomatosis with polyangiitis-like’ orbital pseudotumour. It was exquisitely steroid responsive; mycophenolate mofetil was added from an early stage of treatment.

The patient was referred to rheumatology 3 years after diagnosis as she was unable to reduce her prednisolone below 5mg daily. Her immunology screen was negative. Although her plasma IgG4 levels were normal at 0.14g/L, the original histology was reviewed and immunostained for IgG4. This demonstrated more than 80% of plasma cells to be IgG4 positive, with the previously noted dense fibrosis and chronic inflammatory infiltrate. Her diagnosis was revised to orbital IgG4-RD. She commenced azathioprine as she was planning a pregnancy.

Following an uncomplicated pregnancy in 2014, her symptoms significantly deteriorated, with worsening pain, discharge and swelling in her left eye. A repeat MRI scan showed a new cuff of abnormal tissue within the lateral aspect of the left orbit. She was referred to ophthalmology and underwent left anterior orbitotomy with debulking. Histopathology at this time was consistent with extranodal marginal zone lymphoma (EMZL) of MALT (mucosa-associated lymphoid tissue) type, IgG4 positive. An extraordinary feature was an abundance of crystal-storing histiocytes, presumed to contain ingested IgG4.

To investigate whether she had an IgG4-secreting lymphoma from the beginning or whether there had been malignant transformation of her orbital IgG4-RD, the histology from 2009 was reviewed. This was considered to indicate a MALT-type EMZL.

The patient was referred to haematology and she was treated with localised radiotherapy with an excellent response. She remains in remission following this and she has subsequently been discharged from haematology follow-up.

**Case report - Discussion:**

IgG4-RD is a condition involving fibroinflammatory lesions that can affect any organ in the body. As a newly recognised condition, the role of IgG4 in IgG4-RD is still not fully understood. Questions that remain unanswered include whether or not IgG4 is directly pathogenic and whether there is an increased risk of malignancy in patients with IgG4-RD.

Our patient’s case demonstrates how IgG4-positive lymphoma can mimic IgG4-RD. It is important to keep a broad differential diagnosis in complex cases, and to support the eventual diagnosis with blood results, imaging studies and histology. It is essential to involve colleagues in radiology and histopathology so that a multidisciplinary decision can be made with regards to management. IgG4-RD is a steroid-responsive condition. When there is poor response to conventional treatment, this should raise doubts regarding the diagnosis, and it would be prudent to consider a repeat biopsy as well as obtaining previous pathology specimens so that they can be re-reviewed.

Ocular adnexal lymphomas form 1—2% of non-Hodgkin lymphomas and 8% of extranodal lymphomas. Over recent decades the incidence has increased, partly reflecting better diagnostic techniques. The most common subtype of ocular adnexal lymphomas are EMZL of MALT type, as in our patient.

There are several case reports of lymphoma arising in patients with established IgG4-RD, raising the question of potential lymphomagenesis in IgG4-RD. The earliest reports come from Asia and most have been reported in patients with ocular IgG4-RD. However, the link between malignancy and IgG4-RD is still being investigated. It is important to consider a potential malignancy when managing patients with IgG4-RD, especially if there is a lack of response to steroids.

**Case report - Key learning points:**

1. There is no single diagnostic test for IgG4-RD. Diagnosis should be based on the overall clinical picture, supported by blood results, imaging and histopathology.
2. IgG4-RD is a steroid-responsive disease. If there is a poor response to treatment, the diagnosis should be re-considered.
3. Re-examination of previous biopsy specimens can be invaluable, especially in patients refractory to standard treatment.
4. The link between IgG4-RD and malignancy has not been established and it is important to remain vigilant. As ocular adnexal lymphomas are not uncommon, it is important to keep this differential diagnosis in mind when reviewing patients with eye swelling thought to be secondary to IgG4-related ocular disease.

